# Metastatic squamous cell carcinoma in a cervical lymph node

**DOI:** 10.1002/ccr3.3326

**Published:** 2020-09-11

**Authors:** Zachary W. Bloomer, Andrea N. Snitchler, Mohamed K. M. Shakir, Thanh D. Hoang

**Affiliations:** ^1^ Division of Endocrinology Department of Medicine Walter Reed National Military Medical Center Bethesda Maryland USA; ^2^ Department of Pathology Walter Reed National Military Medical Center Bethesda Maryland USA

**Keywords:** cervical lymphadenopathy, malignancy, neck ultrasound

## Abstract

Cervical lymphadenopathy is a very common complaint for patients presenting to an endocrinology clinic. This case highlights common locations that malignancy presents at as well as their ultrasound characteristics.

## Graphical abstract

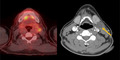

## 1 CLINICAL VIGNETTE

**FIGURE 1:**
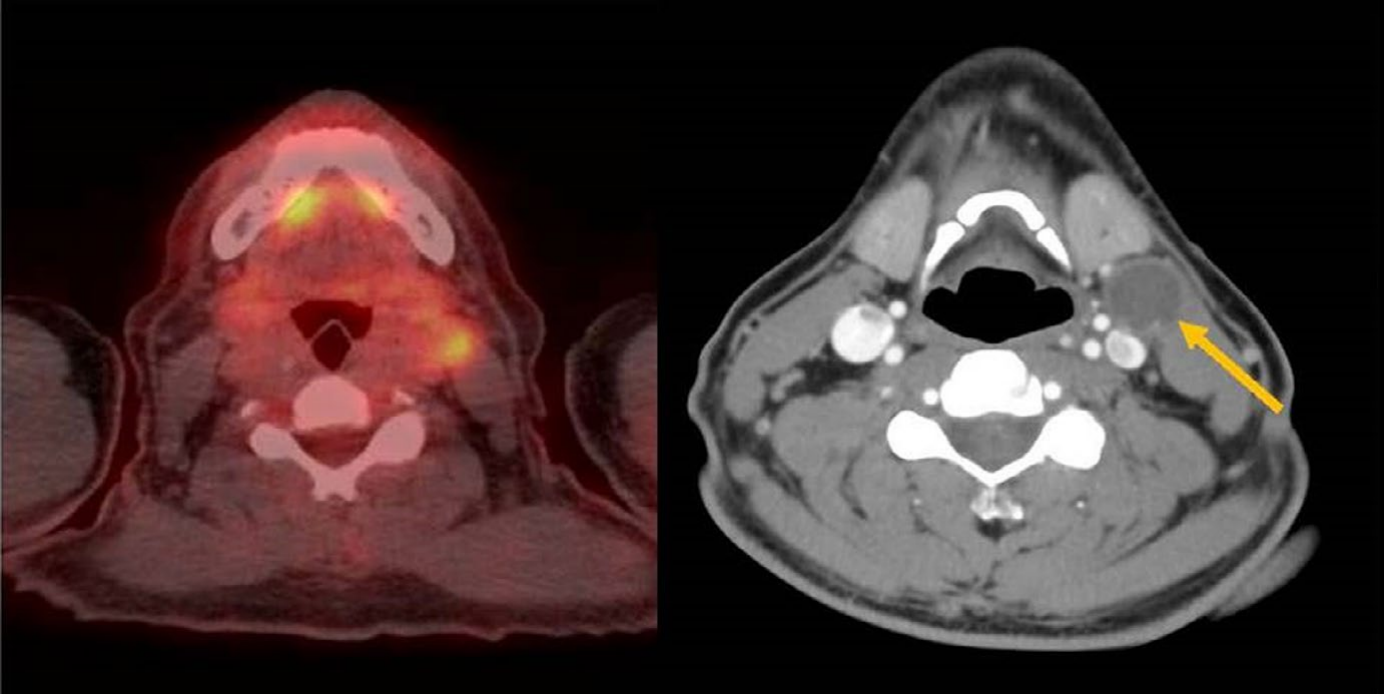
PET/CT scan showing mild increased uptake in the 3.2‐cm cervical node

**FIGURE 2:**
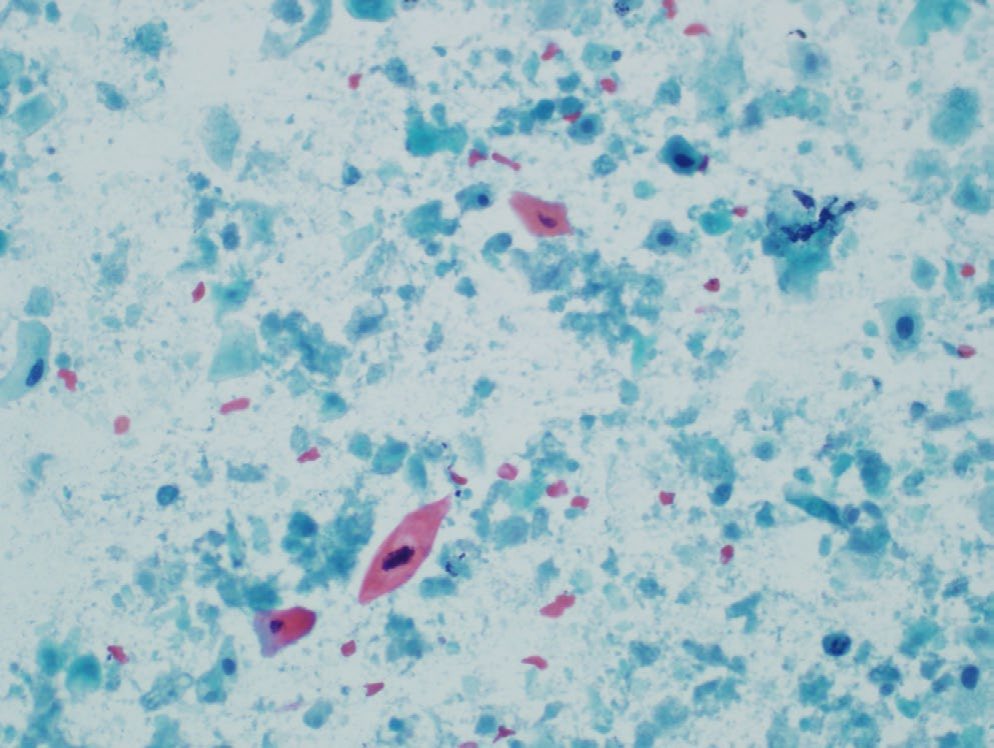
Fine‐needle aspiration (FNA) biopsy of the lymph node showed necrotic debris with cells characterized by dysplastic nuclear features, nuclear membrane irregularities, hyperchromasia, and positive keratin stain. Thyroglobulin of FNA was negative

A 52‐year‐old man presented after a 5‐week history of unilateral painful neck swelling. He denied any fevers, flu‐like symptoms, dysphagia, nor B symptoms. He denied history of smoking or alcohol use. His maternal grandmother had lymphoma but no other family history of cancer. On examination, his vital signs were stable, and there was a nontender left‐sided 2A node easily palpable but no tonsillar enlargement/drainage, no masses noted on the tongue, and no fluid behind either tympanic membrane. Flexible laryngoscopy was performed without obvious abnormalities. Bedside ultrasound revealed a 3.2‐cm cystic appearing nodule without microcalcifications, which was confirmed on CT. No thyroid nodules or other enlarged lymph nodes were seen on imaging. PET scan showed only mild increased uptake in the lymph node and no other abnormal areas of increased uptake (Figure 1). Fine‐needle aspiration biopsy showed necrotic debris with cells characterized by dysplastic nuclear features, nuclear membrane irregularities, hyperchromasia, and positive keratin stain. Thyroglobulin of FNA was negative (Figure 2). These findings are consistent with oropharyngeal squamous cell carcinoma (OPSCC) of the neck.

Oropharyngeal squamous cell carcinoma accounts for 15%‐30% of head and neck cancer.^1^ They typically arise from the base of the tongue or the tonsils. The tumor commonly spreads to level 2 cervical lymph nodes. Biopsy will show atypical cells with darker appearing nuclei, positive keratin, pleomorphic cells, and necrosis. The presence of human papillomavirus may affect management and prognosis. On imaging, these lymph nodes will commonly have cystic features and microcalcifications.^2^ Metastatic papillary thyroid cancer more commonly spreads to level 3‐5 lymph nodes, rather than level 2, but will have similar ultrasound characteristics.^2^ FNA biopsy is the appropriate method of choice in the initial diagnosis as an open biopsy can lead to tumor seeding. Lymph nodes that undergo an inappropriate open biopsy may require a much more aggressive surgery than those that were biopsied via FNA.^3^

This case highlights the high prevalence of squamous cell carcinoma in cervical lymph nodes and their common ultrasound characteristics and location.

## CONFLICT OF INTEREST

None declared.

## AUTHOR'S CONTRIBUTION

ZB: served as the author. MKMS: served as a reviewer. ANS: served as a reviewer and pathologist who provided the slides. TDH: served as a reviewer and corresponding author.

## ETHICAL APPROVAL

The manuscript has been reviewed and approved by the IRB and Public Affairs Office.
